# Unsupervised Learning of Visual Features through Spike Timing Dependent Plasticity

**DOI:** 10.1371/journal.pcbi.0030031

**Published:** 2007-02-16

**Authors:** Timothée Masquelier, Simon J Thorpe

**Affiliations:** 1 Centre de Recherche Cerveau et Cognition, Centre National de la Recherche Scientifique, Université Paul Sabatier, Faculté de Médecine de Rangueil, Toulouse, France; 2 SpikeNet Technology SARL, Labege, France; University College London, United Kingdom

## Abstract

Spike timing dependent plasticity (STDP) is a learning rule that modifies synaptic strength as a function of the relative timing of pre- and postsynaptic spikes. When a neuron is repeatedly presented with similar inputs, STDP is known to have the effect of concentrating high synaptic weights on afferents that systematically fire early, while postsynaptic spike latencies decrease. Here we use this learning rule in an asynchronous feedforward spiking neural network that mimics the ventral visual pathway and shows that when the network is presented with natural images, selectivity to intermediate-complexity visual features emerges. Those features, which correspond to prototypical patterns that are both salient and consistently present in the images, are highly informative and enable robust object recognition, as demonstrated on various classification tasks. Taken together, these results show that temporal codes may be a key to understanding the phenomenal processing speed achieved by the visual system and that STDP can lead to fast and selective responses.

## Author Summary

The paper describes a new biologically plausible mechanism for generating intermediate-level visual representations using an unsupervised learning scheme. These representations can then be used very effectively to perform categorization tasks using natural images. While the basic hierarchical architecture of the system is fairly similar to a number of other recent proposals, the key differences lie in the level of description that is used—individual neurons and spikes—and in the sort of coding scheme involved. Essentially, we have found that a combination of a temporal coding scheme where the most strongly activated neurons fire first with spike timing dependent plasticity leads to a situation where neurons in higher order visual areas will gradually become selective to frequently occurring feature combinations. At the same time, their responses become more and more rapid. We firmly believe that such mechanisms are a key to understanding the remarkable efficiency of the primate visual system.

## Introduction

Temporal constraints pose a major challenge to models of object recognition in cortex. When two images are simultaneously flashed to the left and right of fixation, human subjects can make reliable saccades to the side where there is a target animal in as little as 120–130 ms [1]. If we allow 20–30 ms for motor delays in the oculomotor system, this implies that the underlying visual processing can be done in 100 ms or less. In monkeys, recent recordings from inferotemporal cortex (IT) showed that spike counts over time bins as small as 12.5 ms (which produce essentially a binary vector with either ones or zeros) and only about 100 ms after stimulus onset contain remarkably accurate information about the nature of a visual stimulus [2]. This sort of rapid processing presumably depends on the ability of the visual system to learn to recognize familiar visual forms in an unsupervised manner. Exactly how this learning occurs constitutes a major challenge for theoretical neuroscience. Here we explored the capacity of simple feedforward network architectures that have two key features. First, when stimulated with a flashed visual stimulus, the neurons in the various layers of the system fire asynchronously, with the most strongly activated neurons firing first—a mechanism that has been shown to efficiently encode image information [3]. Second, neurons at later stages of the system implement spike timing dependent plasticity (STDP), which is known to have the effect of concentrating high synaptic weights on afferents that systematically fire early [4,5]. We demonstrate that when such a hierarchical system is repeatedly presented with natural images, these intermediate-level neurons will naturally become selective to patterns that are reliably present in the input, while their latencies decrease, leading to both fast and informative responses. This process occurs in an entirely unsupervised way, but we then show that these intermediate features are able to support categorization.

**Figure 1:**
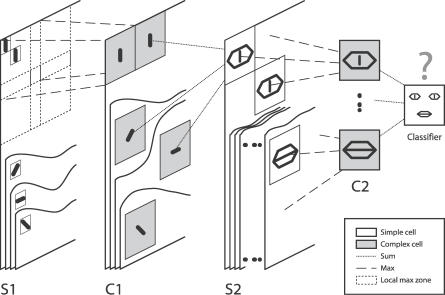
Overview of the Five-Layer Feedforward Spiking Neural Network As in HMAX [7], we alternate simple cells that gain selectivity through a sum operation, and complex cells that gain shift and scale invariance through a max operation (which simply consists of propagating the first received spike). Cells are organized in retinotopic maps until the S2 layer (inclusive). S1 cells detect edges. C1 maps subsample S1 maps by taking the maximum response over a square neighborhood. S2 cells are selective to intermediate-complexity visual features, defined as a combination of oriented edges (here we symbolically represented an eye detector and a mouth detector). There is one S1–C1–S2 pathway for each processing scale (not represented). Then C2 cells take the maximum response of S2 cells over all positions and scales and are thus shift- and scale-invariant. Finally, a classification is done based on the C2 cells' responses (here we symbolically represented a face/nonface classifier). In the brain, equivalents of S1 cells may be in V1, S2 cells in V1–V2, S2 cells in V4–PIT, C2 cells in AIT, and the final classifier in PFC. This paper focuses on the learning of C1 to S2 synaptic connections through STDP.

Our network belongs to the family of feedforward hierarchical convolutional networks, as in [6–10]. To be precise, its architecture is inspired from Serre, Wolf, and Poggio's model of object recognition [6], a model that itself extends HMAX [7] and performs remarkably well with natural images. Like them, in an attempt to model the increasing complexity and invariance observed along the ventral pathway [11,12], we use a four-layer hierarchy (S1–C1–S2–C2) in which simple cells (S) gain their selectivity from a linear sum operation, while complex cells (C) gain invariance from a nonlinear max pooling operation (see Figure 1 and Methods for a complete description of our model).

Nevertheless, our network does not only rely on static nonlinearities: it uses spiking neurons and operates in the temporal domain. At each stage, the time to first spike with respect to stimulus onset (or, to be precise, the rank of the first spike in the spike train, as we will see later) is supposed to be the “key variable,” that is, the variable that contains information and that is indeed read out and processed by downstream neurons. When presented with an image, the first layer's S1 cells, emulating V1 simple cells, detect edges with four preferred orientations, and the more strongly a cell is activated, the earlier it fires. This intensity–latency conversion is in accordance with recordings in V1 showing that response latency decreases with the stimulus contrast [13,14] and with the proximity between the stimulus orientation and the cell's preferred orientation [15]. It has already been shown how such orientation selectivity can emerge in V1 by applying STDP on spike trains coming from retinal ON- and OFF-center cells [16], so we started our model from V1 orientation-selective cells. We also limit the number of spikes at this stage by introducing competition between S1 cells through a one-winner-take-all mechanism: at a given location—corresponding to one cortical column—only the spike corresponding to the best matching orientation is propagated (sparsity is thus 25% at this stage). Note that *k*-winner-take-all mechanisms are easy to implement in the temporal domain using inhibitory GABA interneurons [17].

These S1 spikes are then propagated asynchronously through the feedforward network of integrate-and-fire neurons. Note that within this time-to-first-spike framework, the maximum operation of complex cells simply consists of propagating the first spike emitted by a given group of afferents [18]. This can be done efficiently with an integrate-and-fire neuron with low threshold that has synaptic connections from all neurons in the group.

Images are processed one by one, and we limit activity to at most one spike per neuron, that is, only the initial spike wave is propagated. Before presenting a new image, every neuron's potential is reset to zero. We process various scaled versions of the input image (with the same filter size). There is one S1–C1–S2 pathway for each processing scale (not represented on Figure 1). This results in S2 cells with various receptive field sizes (see Methods). Then C2 cells take the maximum response (i.e., first spike) of S2 cells over all positions and scales, leading to position and scale invariant responses.

This paper explains how STDP can set the C1–S2 synaptic connections, leading to intermediate-complexity visual features, whose equivalent in the brain may be in V4 or IT. STDP is a learning rule that modifies the strength of a neuron's synapses as a function of the precise temporal relations between pre- and postsynaptic spikes: an excitatory synapse receiving a spike before a postsynaptic one is emitted is potentiated (long-term potentiation) whereas its strength is weakened the other way around (long-term depression) [19]. The amount of modification depends on the delay between these two events: maximal when pre- and postsynaptic spikes are close together, and the effects gradually decrease and disappear with intervals in excess of a few tens of milliseconds [20–22]. Note that STDP is in agreement with Hebb's postulate because presynaptic neurons that fired slightly before the postsynaptic neuron are those that “took part in firing it.” Here we used a simplified STDP rule where the weight modification does not depend on the delay between pre- and postsynaptic spikes, and the time window is supposed to cover the whole spike wave (see Methods). We also use 0 and 1 as “soft bounds” (see Methods), ensuring the synapses remain excitatory. Several authors have studied the effect of STDP with Poisson spike trains [4,23]. Here, we demonstrate STDP's remarkable ability to detect statistical regularities in terms of earliest firing afferent patterns within visual spike trains, despite their very high dimensionality inherent to natural images.

Visual stimuli are presented sequentially, and the resulting spike waves are propagated through to the S2 layer, where STDP is used. We use restricted receptive fields (i.e., S2 cells only integrate spikes from an *s* × *s* square neighborhood in the C1 maps corresponding to one given processing scale) and weight-sharing (i.e., each *prototype* S2 cell is duplicated in retinotopic maps and at all scales). Starting with a random weight matrix (size = 4 × *s* × *s*), we present the first visual stimuli. Duplicated cells are all integrating the spike train and compete with each other. If no cell reaches its threshold, nothing happens and we process the next image. Otherwise for each prototype the first duplicate to reach its threshold is the winner. A one-winner-take-all mechanism prevents the other duplicated cells from firing. The winner thus fires and the STDP rule is triggered. Its weight matrix is updated, and the change in weights is duplicated at all positions and scales. This allows the system to learn patterns despite changes in position and size in the training examples. We also use local inhibition between different prototype cells: when a cell fires at a given position and scale, it prevents all other cells from firing later at the same scale and within an *s*/2 × *s*/2 square neighborhood of the firing position. This competition, only used in the learning phase, prevents all the cells from learning the same pattern. Instead, the cell population self-organizes, each cell trying to learn a distinct pattern so as to cover the whole variability of the inputs.

If the stimuli have visual features in common (which should be the case if, for example, they contain similar objects), the STDP process will extract them. That is, for some cells we will observe convergence of the synaptic weights (by saturation), which end up being either close to 0 or to 1. During the convergence process, synapses compete for control of the timing of postsynaptic spikes [4]. The winning synapses are those through which the earliest spikes arrive (on average) [4,5], and this is true even in the presence of jitter and spontaneous activity [5] (although the model presented in this paper is fully deterministic). This “preference” for the earliest spikes is a key point since the earliest spikes, which correspond in our framework to the most salient regions of an image, have been shown to be the most informative [3]. During the learning, the postsynaptic spike latency decreases [4,5,24]. After convergence, the responses become selective (in terms of latency) [5] to visual features of intermediate complexity similar to the features used in earlier work [8]. Features can now be defined as clusters of afferents that are consistently among the earliest to fire. STDP detects these kinds of statistical regularities among the spike trains and creates one unit for each distinct pattern.

## Results

**Figure 2:**
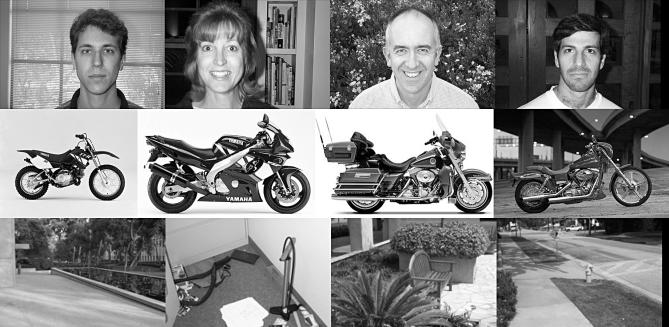
Sample Pictures from the Caltech Datasets The top row shows examples of faces (all unsegmented), the middle row shows examples of motorbikes (some are segmented, others are not), and the bottom row shows examples of distractors.

We evaluated our STDP-based learning algorithm on two California Institute of Technology datasets, one containing faces and the other motorbikes, and a distractor set containing backgrounds, all available at http://www.vision.caltech.edu (see Figure 2 for sample pictures). Note that most of the images are not segmented. Each dataset was split into a training set, used in the learning phase, and a testing set, not seen during the learning phase but used afterward to evaluate the performance on novel images. This standard cross-validation procedure allows the measurement of the system's ability to *generalize,* as opposed to learning the specific training examples. The splits used were the same as Fergus, Perona, and Zisserman [25]. All images were rescaled to be 300 pixels in height (preserving the aspect ratio) and converted to grayscale values.

**Figure 3:**
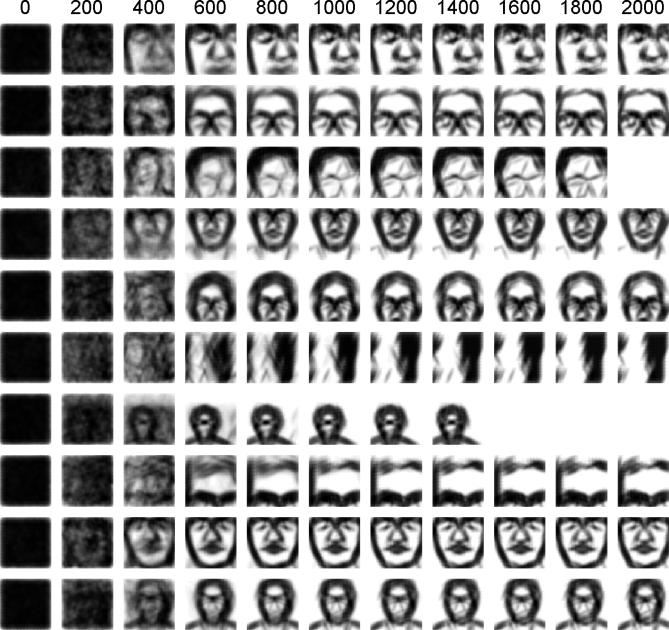
Evolution of Reconstructions for Face Features At the top is the number of postsynaptic spikes emitted. Starting from random preferred stimuli, cells detect statistical regularities among the input visual spike trains after a few hundred discharges and progressively develop selectivity to those patterns. A few hundred more discharges are needed to reach a stable state. Furthermore, the population of cells self-organizes, with each cell effectively trying to learn a distinct pattern so as to cover the whole variability of the inputs.

**Figure 4:**
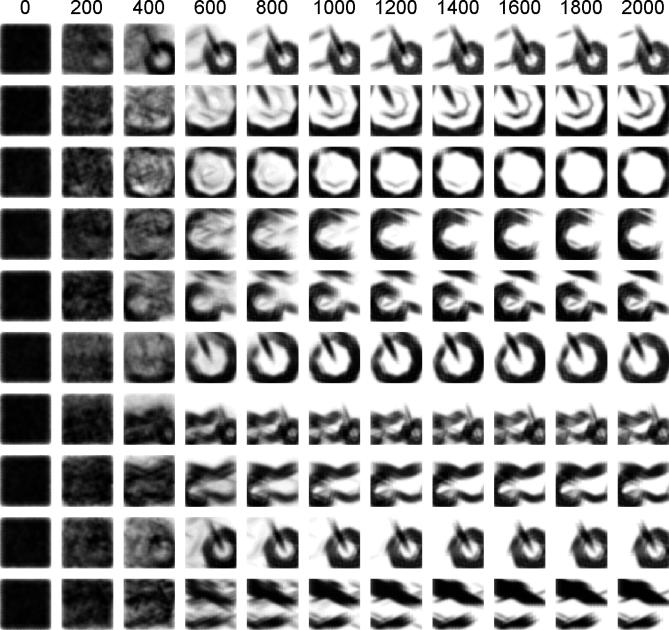
Evolution of Reconstructions for Motorbike Features

We first applied our unsupervised STDP-based algorithm on the face and motorbike training examples (separately), presented in random order, to build two sets of ten class-specific C2 features. Each C2 cell has one preferred input, defined as a combination of edges (represented by C1 cells). Note that many gray-level images may lead to this combination of edges because of the local max operation of C1 cells and because we lose the “polarity” information (i.e., which side of the edge is darker). However, we can reconstruct a representation of the set of preferred images by convolving the weight matrix with a set of kernels representing oriented bars. Since we start with random weight matrices, at the beginning of the learning process the reconstructed preferred stimuli do not make much sense. But as the cells learn, structured representations emerge, and we are usually able to identify the nature of the cells' preferred stimuli. Figures 3 and 4 show the reconstructions at various stages of learning for the face and motorbike datasets, respectively. We stopped the learning after 10,000 presentations.

Then we turned off the STDP rule and tested these STDP-obtained features' ability to support face/nonface and motorbike/nonmotorbike classification. This paper focuses more on feature extraction than on sophisticated classification methods, so we first used a very simple decision rule based on the number of C2 cells that fired with each test image, on which a threshold is applied. Such a mechanism could be easily implemented in the brain. The threshold was set at the equilibrium point (i.e., when the false positive rate equals the missed rate). In Table 1 we report good classification results with this “simple-count” scheme in terms of area under the receiver operator characteristic (ROC) and the performance rate at equilibrium point.

**Table 1:**
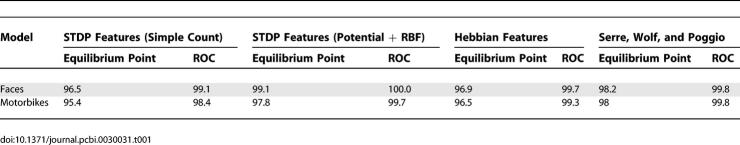
Classification Results

We also evaluated a more complicated classification scheme. C2 cells' thresholds were supposed to be infinite, and we measured the final potentials they reached after having integrated the whole spike train generated by the image. This final potential can be seen as the number of early spikes in common between a current input and a stored prototype (this contrasts with HMAX and extensions [6,7,26], where a Euclidian distance or a normalized dot product is used to measure the difference between a stored prototype and a current input). Note that this potential is contrast invariant: a change in contrast will shift all the latencies but will preserve the spike order. The final potentials reached with the training examples were used to train a radial basis function (RBF) classifier (see Methods). We chose this classifier because linear combination of Gaussian-tuned units is hypothesized to be a key mechanism for generalization in the visual system [27]. We then evaluated the RBF on the testing sets. As can be seen in Table 1, performance with this “potential + RBF” scheme was better.

Using only ten STDP-learnt features, we reached on those two classes a performance that is comparable to that of Serre, Wolf, and Poggio's model, which itself is close to the best state-of-the-art computer vision systems [6]. However, their system is more generic. Classes with more intraclass variability (for example, animals) appear to pose a problem with our approach because a lot of training examples (say a few tens) of a given feature type are needed for the STDP process to learn it properly.

Our approach leads to the extraction of a small set (here ten) of highly informative class-specific features. This is in contrast with Serre et al.'s approach where many more (usually about a thousand) features are used. Their sets are more generic and are suitable for many different classes [6]. They rely on the final classifier to “select” diagnostic features and appropriately weight them for a given classification task. Here, STDP will naturally focus on what is common to the positive training set, that is, target object features. The background is generally not learned (at least not in priority), since backgrounds are almost always too different from one image to another for the STDP process to converge. Thus, we directly extract diagnostic features, and we can obtain reasonably good classification results using only a threshold on the number of detected features. Furthermore, as STDP performs vector quantization from multiple examples as opposed to “one-shot learning,” it will not learn the noise, nor anything too specific to a given example, with the result that it will tend to learn archetypical features.

Another key point is the natural trend of the algorithm to learn salient regions, simply because they correspond to the earliest spikes, with the result that neurons whose receptive fields cover salient regions are likely to reach their threshold (and trigger the STDP rule) before neurons “looking” at other regions. This contrasts with more classical competitive learning approaches, where input normalization helps different input patterns to be equally effective in the learning process [28]. Note that “salient” means within our network “with well-defined contrasted edges,” but saliency is a more generic concept of local differences, for example, in intensity, color, or orientations as in the model of Itti, Koch, and Niebur [29]. We could use other types of S1 cells to detect other types of saliency, and, provided we apply the same intensity–latency conversion, STDP would still focus on the most salient regions. Saliency is known to drive attention (see [30] for a review). Our model predicts that it also drives the learning. Future experimental work will test this prediction.

**Figure 5:**
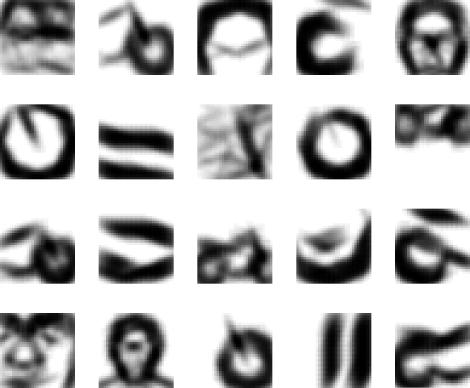
Final Reconstructions for the 20 Features in the Mixed Case The 20 cells self-organized, some having developed selectivity to face features, and some to motorbike features.

Of course, in real life we are unlikely to see many examples of a given category in a row. That is why we performed a second simulation, where 20 C2 cells were presented with the face, motorbike, and background training pictures in random order, and the STDP rule was applied. Figure 5 shows all the reconstructions for this mixed simulation after 20,000 presentations. We see that the 20 cells self-organized, some of them having developed selectivity to face features, and others to motorbike features. Interestingly, during the learning process the cells rapidly showed a preference for one category. After a certain degree of selectivity had been reached, the face-feature learning was not influenced by the presentation of motorbikes (and vice versa), simply because face cells will not fire (and trigger the STDP rule) on motorbikes. Again we tested the quality of these features with a (multiclass) classification task, using an RBF network and a “one-versus-all” approach (see Methods). As before, we tested two implementations: one based on “binary detections + RBF” and one based on “potential + RBF”. Note that a simple detection count cannot work here, as we need at least some supervised learning to know which feature (or feature combination) is diagnostic (or antidiagnostic) of which class. Table 2 shows the confusion matrices obtained on the testing sets for both implementations, leading, respectively, to 95.0% and 97.7% of correct classifications on average. It is worth mentioning that the “potential + RBF” system perfectly discriminated between faces and motorbikes—although both were presented in the unsupervised STDP-based learning phase.

**Table 2:**
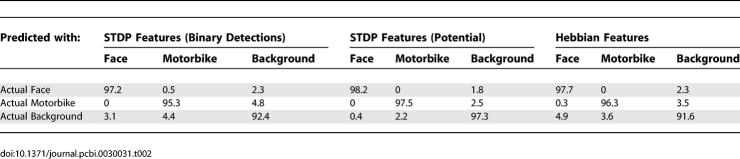
Confusion Matrices

A third type of simulation was run to illustrate the STDP learning process. For these simulations, only three C2 cells and four processing scales (71%, 50%, 35%, and 25%) were used. We let at most one cell fire at each processing scale. The rest of the parameters were strictly identical to the other simulations (see Methods). Videos S1–S3 illustrate the STDP learning process with, respectively, faces, motorbikes, and a mix of faces, motorbikes, and background pictures. It can be seen that after convergence the STDP feature showed a good tradeoff between selectivity (very few false alarms) and invariance (most of the targets were recognized).

An interesting control is to compare the STDP learning rule with a more standard hebbian rule in this precise framework. For this purpose, we converted the spike trains coming from C1 cells into a vector of (real-valued) C1 activities *X*_C1_, supposed to correspond to firing rates (see Methods). Each S2 cell was no longer modeled at the integrate-and-fire level but was supposed to respond with a (static) firing rate *Y*_S2_ given by the normalized dot product:

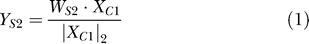
where *W*_S2_ is the synaptic weight vector of the S2 cell (see Methods).

The S2 cells still competed with each other, but the *k*-winner-take-all mechanisms now selected the cells with the highest firing rates (instead of the first one to fire). Only the cells whose firing rates reached a certain threshold were considered in the competition (see Methods). The winners now triggered the following modified hebbian rule (instead of STDP):

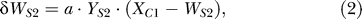
where a decay term has been added to keep the weight vector bounded (however, the rule is still local, unlike an explicit weight normalization). Note that this precaution was not needed in the STDP case because competition between synapse naturally bounds the weight vector [4]. The rest of the network is strictly identical to the STDP case.

**Figure 6:**
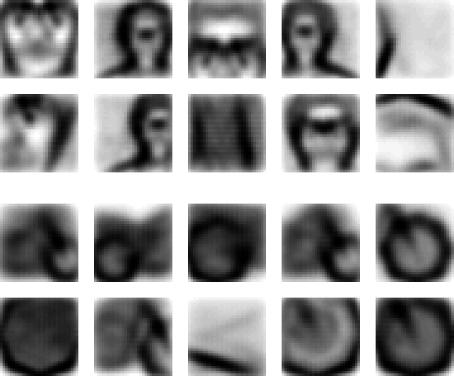
Hebbian Learning (Top) Final reconstructions for the ten face features. (Bottom) The ten motorbike features.

**Figure 7:**
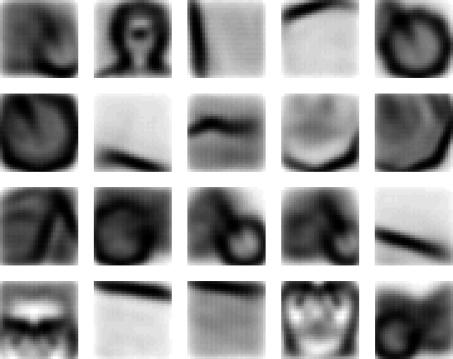
Hebbian Learning: Final Reconstructions for the 20 Features in the Mixed Case As with STDP-based learning, the 20 cells self-organized, some having developed selectivity to face features, and some to motorbike features.

Figure 6 shows the reconstruction of the preferred stimuli for the ten C2 cells after 10,000 presentations for the face stimuli (Figure 6, top) and the motorbikes stimuli (Figure 6, top). Again we can usually recognize the face and motorbike parts to which the cells became selective (even though the reconstructions look fuzzier than in the STDP case because the final weights are more graded). We also tested the ability of these hebbian-obtained features to support face/nonface and motorbike/nonmotorbike classification once fed into an RBF, and the results are shown in Table 1 (last column). We also evaluated the hebbian features with the multiclass setup. Twenty cells were presented with the same mix of face, motorbike, and background pictures as before. Figure 7 shows the final reconstructions after 20,000 presentations, and Table 2 shows the confusion matrix (last columns).

The main conclusion is that the modified hebbian rule is also able to extract pertinent features for classification (although performance on these tests appears to be slightly worse). This is not very surprising as STDP can be seen as a hebbian rule transposed in the temporal domain, but it was worth checking. Where STDP would detect (and create selectivity to) sets of units that are consistently among the first one to fire, the hebbian rule detects (and creates selectivity to) sets of units that consistently have the highest firing rates. However, we believe the temporal framework is a better description of what really happens at the neuronal level, at least in ultrarapid categorization tasks. Furthermore, STDP also explains how the system becomes faster and faster with training, since the neurons learn to decode the first information available at their afferents' level (see also Discussion).

## Discussion

While the ability of hierarchical feedforward networks to support classification is now reasonably well established (e.g., [6–8,10]), how intermediate-complexity features can be learned remains an open problem, especially with cluttered images. In the original HMAX model, S2 features were not learned but were manually hardwired [7]. Later versions used huge sets of random crops (say 1,000) taken from natural images and used these crops to “imprint” S2 cells [6]. This approach works well but is costly since redundancy is very high between features, and many features are irrelevant for most (if not all) of the tasks. To select only pertinent features for a given task, Ullman proposed an interesting criterion based on mutual information [8], leaving the question of possible neural implementation open. LeCun showed how visual features in a convolutional network could be learned in a supervised manner using back-propagation [10], without claiming this algorithm was biologically plausible. Although we may occasionally use supervised learning to create a set of features suitable for a particular recognition task, it seems unrealistic that we need to do that each time we learn a new class. Here we took another approach: one layer with unsupervised competitive learning is used as input for a second layer with supervised learning. Note that this kind of hybrid scheme has been found to learn much faster than a two-layer backpropagation network [28].

Our approach is a bottom-up one: instead of intuiting good image-processing schemes and discussing their eventual neural correlates, we took known biological phenomena that occur at the neuronal level, namely integrate-and-fire and STDP, and observed where they could lead at a more integrated level. The role of the simulations with natural images is thus to provide a “plausibility proof” that such mechanisms could be implemented in the brain.

However, we have made four main simplifications. The first one was to propagate input stimuli one by one. This may correspond to what happens when an image is flashed in an ultrarapid categorization paradigm [1], but normal visual perception is an ongoing process. However, every 200 ms or 300 ms we typically perform a saccade. The processing of each of these discrete “chunks” seems to be optimized for rapid execution [31], and we suggest that much can be done with the feedforward propagation of a single spike wave. Furthermore, even when fixating, our eyes are continuously making microsaccades that could again result in repetitive waves of activation. This idea is in accordance with electrophysiological recordings showing that V1 neuron activity is correlated with microsaccades [32]. Here we assumed the successive waves did not interfere, which does not seem too unreasonable given that the neuronal time constants (integration, leak, STDP window) are in the range of a few tens of milliseconds whereas the interval between saccades and microsaccades is substantially longer. It is also possible that extraretinal signals suppress interference by shutting down any remaining activity before propagating the next wave. Note that this simplification allows us to use nonleaky integrate-and-fire neurons and an infinite STDP time window. More generally, as proposed by Hopfield [33], waves could be generated by population oscillations that would fire one cell at a time in advance of the maximum of the oscillation, which increases with the inputs the cell received. This idea is in accordance with recordings in area 17 of cat visual cortex showing that suboptimal cells reveal a systematic phase lag relative to optimally stimulated cells [34].

The second simplification we have made is to use only five layers (including the classification layer), whereas processing in the ventral stream involves many more layers (probably about ten), and complexity increases more slowly than suggested here. However, STDP as a way to combine simple features into more complex representations, based on statistical regularities among earliest spike patterns, seems to be a very efficient learning rule and could be involved at all stages.

The third main simplification we have made consists of using restricted receptive fields and weight sharing, as do most of the bio-inspired hierarchical networks [6–10] (networks using these techniques are called *convolutional networks*). We built shift and scale invariance by structure (and not by training) by duplicating S1, C1, and S2 cells at all positions and scales. This is a way to reduce the number of free parameters (and therefore the VC dimension [35]) of the network by incorporating prior information into the network design: responses should be scale- and shift-invariant. This greatly reduces the number of training examples needed. Note that this technique of weight sharing could be applied to other transformations than shifting and scaling, for instance, rotation and symmetry. However, it is difficult to believe that the brain could really use weight sharing since, as noted by Földiák [36], updating the weights of all the simple units connected to the same complex unit is a nonlocal operation. Instead, he suggested that at least the low-level features could be learned locally and independently. Subsequently, cells with similar preferred stimulus may connect adaptively to the same complex cell, possibly by detecting correlation across time thanks to a trace rule [36]. Wallis, Rolls, and Milward successfully implemented this sort of mechanism in a multilayered hierarchical network called Vis-Net [37,38]; however, performance after learning objects from unsegmented natural images was poor [39]. Future work will evaluate the use of local learning and adaptative complex pooling in our network, instead of exact weight sharing. Learning will be much slower but should lead to similar STDP features. Note that it seems that monkeys can recognize high-level objects at scales and positions that have not been experienced previously [2,40]. It could be that in the brain local learning and adaptative complex pooling are used up to a certain level of complexity, but not for high-level objects. These high-level objects could be represented with a combination of simpler features that would already be shift- and scale-invariant. As a result, there would be less need for spatially specific representations for high-level objects.

The last main simplification we have made is to ignore both feedback loops and top-down influences. While normal, everyday vision extensively uses feedback loops, the temporal constraints almost certainly rule them out in an ultrarapid categorization task [41]. The same cannot be said about the top-down signals, which do not depend directly on inputs. For example, there is experimental evidence that the selectivity to the “relevant” features for a given recognition task can be enhanced in IT [42] and in V4 [43], possibly thanks to a top-down signal coming from the prefrontal cortex, thought to be involved in the categorization process. These effects, for example, modeled by Szabo et al. [44], are not taken into account here.

Despite these four simplifications, we think our model captures two key mechanisms used by the visual system for rapid object recognition. The first one is the importance of the first spikes for rapidly encoding the most important information about a visual stimulus. Given the number of stages involved in high-level recognition and the short latencies of selective responses recorded in monkeys' IT [2], the time window available for each neuron to perform its computation is probably about 10–20 ms [45] and will rarely contain more than one or two spikes. The only thing that matters for a neuron is whether an afferent fires early enough so that the presynaptic spike falls in the critical time window, while later spikes cannot be used for ultrarapid categorization. At this point (but only at this point), we have to consider two hypotheses: either presynaptic spike times are completely stochastic (for example, drawn from a Poisson distribution), or they are somewhat reliable. The first hypothesis causes problems since the first presynaptic spikes (again the only ones taken into account) will correspond to a subset of the afferents that is essentially random, and will not contain much information about their real activities [46]. A solution to this problem is to use populations of redundant neurons (with similar selectivity) to ensure the first presynaptic spikes do correspond on average to the most active populations of afferents. In this work we took the second hypothesis, assuming the time to first spike of the afferents (or, to be precise, their firing order) was reliable and did reflect a level of activity. This second hypothesis receives experimental support. For example, recent recordings in monkeys show that IT neurons' responses in terms of spike count *close to stimulus onset* (100–150 ms time bin) seem to be too reliable to be fit by a typical Poisson firing rate model [47]. Another recent electrophysiological study in monkeys showed that IT cell's latencies do contain information about the nature of a visual stimulus [48]. There is also experimental evidence for precise spike time responses in V1 and in many other neuronal systems (see [49] for a review).

Very interestingly, STDP provides an efficient way to develop selectivity to first spike patterns, as shown in this work. After convergence, the potential reached by an STDP neuron is linked to the number of early spikes in common between the current input and a stored prototype. This “early spike” versus “later spike” neural code (while the spike order within each bin does not matter) has not only been proven robust enough to perform object recognition in natural images but is fast to read out: an accurate response can be produced when only the earliest afferents have fired. The use of such a mechanism at each stage of the ventral stream could account for the phenomenal processing speed achieved by the visual system.

## Materials and Methods

Here is a detailed description of the network, the STDP model, and the classification methods.

### S1 cells.

S1 cells detect edges by performing a convolution on the input images. We are using 5 × 5 convolution kernels, which roughly correspond to Gabor filters with wavelength of 5 (i.e., the kernel contains one period), effective width 2, and four preferred orientations: π/8, π/4 + π/8, π/2 + π/8, and 3π/4 + π/8 (π/8 is there to avoid focusing on horizontal and vertical edges, which are seldom diagnostic). We apply those filters to five scaled versions of the original image: 100%, 71%, 50%, 35%, and 25%. There are thus 4 × 5 = 20 S1 maps. S1 cells emit spikes with a latency that is inversely proportional to the absolute value of the convolution (the response is thus invariant to an image negative operation). We also limit activity at this stage: at a given processing scale and location, only the spike corresponding to the best matching orientation is propagated.

### C1 cells.

C1 cells propagate the first spike emitted by S1 cells in a 7 × 7 square of a given S1 map (which corresponds to one preferred orientation and one processing scale). Two adjacent C1 cells in a C1 map correspond to two 7 × 7 squares of S1 cells shifted by six S1 cells (and thus overlap of one S1 row). C1 maps thus subsample S1 maps. To be precise, neglecting the side effects, there are 6 × 6 = 36 times fewer C1 cells than S1 cells. As proposed by Riesenhuber and Poggio [7], this maximum operation is a biologically plausible way to gain local shift invariance. From an image processing point of view, it is a way to perform subsampling within retinotopic maps without flattening high spatial frequency peaks (as would be the case with local averaging).

We also use a local lateral inhibition mechanism at this stage: when a C1 cell emits a spike, it increases the latency of its neighbors within an 11 × 11 square in the map with the same preferred orientation and the same scale. The percentage of latency increase decreases linearly with the distance from the spike, from 15% to 5%. As a result, if a region is clearly dominated by one orientation, cells will inhibit each other and the spike train will be globally late and thus unlikely to be “selected” by STDP.

### S2 cells.

S2 cells correspond to intermediate-complexity visual features. Here we used ten prototype S2 cell types, and 20 in the mixed simulation. Each prototype cell is duplicated in five maps (weight sharing), each map corresponding to one processing scale. Within those maps, the S2 cells can integrate spikes only from the four C1 maps of the corresponding processing scale. The receptive field size is 16 × 16 C1 cells (neglecting the side effects; this leads to 96 × 96 S1 cells, and the corresponding receptive field size in the original image is [96 / processing scale]^2^). C1–S2 synaptic connections are set by STDP.

Note that we did not use a leakage term. In the brain, by progressively resetting membrane potentials toward their resting levels, leakiness will decrease the interference between two successive spike waves. In our model we process spike waves one by one and reset all the potentials before each propagation, and so leaks are not needed.

Finally, activity is limited at this stage: a *k*-winner-take-all strategy ensures at most two cells that can fire for each processing scale. This mechanism, only used in the learning phase, helps the cells to learn patterns with different real sizes. Without it, there is a natural bias toward “small” patterns (i.e., large scales), simply because corresponding maps are larger, and so likeliness of firing with random weights at the beginning of the STDP process is higher.

### C2 cells.

Those cells take for each prototype the maximum response (i.e., first spike) of corresponding S2 cells over all positions and processing scales, leading to ten shift- and scale-invariant cells (20 in the mixed case).

### STDP model.

We used a simplified STDP rule:

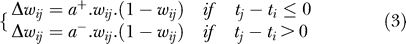
where *i* and *j* refer, respectively, to the post- and presynaptic neurons, *t_i_* and *t_j_* are the corresponding spike times, Δ*w_ij_* is the synaptic weight modification, and *a* ^+^ and *a* ^−^ are two parameters specifying the amount of change. Note that the weight change does not depend on the exact *t_i_* − *t_j_* value, but only on its sign. We also used an infinite time window. These simplifications are equivalent to assuming that the intensity–latency conversion of S1 cells compresses the whole spike wave in a relatively short time interval (say, 20–30 ms), so that all presynaptic spikes necessarily fall close to the postsynaptic spike time, and the change decrease becomes negligible. In the brain, this change decrease and the limited time window are crucial: they prevent different spike waves coming from different stimuli from interfering in the learning process. In our model, we propagate stimuli one by one, so these mechanisms are not needed. Note that with this simplified STDP rule only the *order* of the spikes matters, not their precise timings. As a result, the intensity–latency conversion function of S1 cells has no impact, and any monotonously decreasing function gives the same results.

The multiplicative term *w_ij_* · (1 − *w_ij_*) ensures the weight remains in the range [0,1] (excitatory synapses) and implements a soft bound effect: when the weight approaches a bound, weight changes tend toward zero.

We also applied long-term depression to synapses through which no presynaptic spike arrived, exactly as if a presynaptic spike had arrived after the postsynaptic one. This is useful to eliminate the noise due to original random weights on synapses through which presynaptic spikes never arrive.

 As the STDP learning progresses, we increase *a* ^+^ and 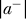 To be precise, we start with *a* ^+^ = 2^−6^ and multiply the value by 2 every 400 postsynaptic spikes, until a maximum value of 2^−2^. *a* ^−^ is adjusted so as to keep a fixed *a* ^+^/*a* ^−^ ratio (−4/3). This allows us to accelerate convergence when the preferred stimulus is somewhat “locked,” whereas directly using high learning rates with the random initial weights leads to erratic results.

We used a threshold of 64 (= 1/4 × 16 × 16). Initial weights are randomly generated, with mean 0.8 and standard deviation 0.05.

### Classification setup.

We used an RBF network. In the brain, this classification step may be done in the PFC using the outputs of IT. Let *X* be the vector of C2 responses (containing either binary detections with the first implementation or final potentials with the second one). This kind of classifier computes an expression of the form:

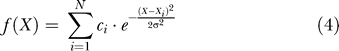
and then classifies based on whether or not *f*(*X*) reaches a threshold. Supervised learning at this stage involves adjusting the synaptic weights *c* so as to minimize a (regularized) error on the training set [27]. The *X_i_* correspond to C2 responses for some training examples (1/4 of the training set randomly selected). The full training set was used to learn the *c_i_*. We used *σ* = 2 and *λ* = 10^−12^ (regularization parameter).

The multiclass case was handled with a “one-versus-all approach.” If *n* is the number of classes (here, three), *n* RBF classifiers of the kind “class I” versus “all other classes” are trained. At the time of testing, each one of the *n* classifiers emits a (real-valued) prediction that is linked to the probability of the image belonging to its category. The assigned category is the one that corresponds to the highest prediction value.

### Hebbian learning.

The spike trains coming from C1 cells were converted into real-valued activities (supposed to correspond to firing rates) by taking the inverse of the first spikes' latencies (note that these activities do not correspond exactly to the convolution values because of the local lateral inhibition mechanism of layer C1). The activities (or firing rates) of S2 units were computed as:

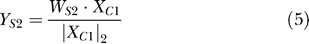
where *W*_S2_ is the synaptic weight vector of the S2 cell. Note that the normalization causes an S2 cell to respond maximally when the input vector *X*_C1_ is collinear to its weight vector *W*_S2_ (neural circuits for such normalization have been proposed in [27]). Hence *W*_S2_ (or any vector collinear to it) is the preferred stimulus of the S2 cell. With another stimulus *X*_C1_ the response is proportional to the cosine between *W*_S2_ and *X*_C1_. This kind of tuning has been used in extensions of HMAX [26]. It is similar to the Gaussian tuning of the original HMAX [7], but it is invariant to the norm of the input (i.e., multiplying the input activities by 2 has no effect on the response), which allows us to remain contrast-invariant (see also [26] for a comparison between the two kinds of tuning).

Only the cells whose activities were above a threshold were considered in the competition process. It was found useful to use individual adaptative thresholds: each time a cell was among the winners, its threshold was set to 0.91 times its activity (this value was tuned to get approximately the same number of weight updates as with STDP). The competition mechanism was exactly the same as before, except that it selected the most active units and not the first one to fire. The winners' weight vectors were updated with the following modified hebbian rule:

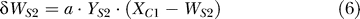



*a* is the learning rate. It was found useful to start with a small learning rate (0.002) and to geometrically increase it every ten iterations. The geometric ratio was set to reach a learning rate of 0.02 after 2,000 iterations, after which the learning rate stayed constant.

### Differences from the model of Serre, Wolf, and Poggio.

Here we summarize the differences between our model and their model [6] in terms of architecture (leaving the questions of learning and temporal code aside).

We process various scaled versions of the input image (with the same filter size), instead of using various filter sizes on the original image: S1 level, only the best matching orientation is propagated; C1 level, we use lateral inhibition (see above); S2 level, the similarity between a current input and the stored prototype is linked to the number of early spikes in common between the corresponding spike trains, while Serre et al. use the Euclidian distance between the corresponding patches of C1 activities.

We used an RBF network and not a Support Vector Machine.

## Supporting Information

Video S1 Face-Feature Learning Here we presented the face-training examples in random order, propagated the corresponding spike waves, and applied the STDP rule. At the top of the screen, the input image is shown, with red, green, or blue squares indicating the receptive fields of the cells that fired (if any). At the bottom of the screen, we reconstructed the preferred stimuli of the three C2 cells. Above each reconstruction, the number of postsynaptic spikes emitted is shown with the corresponding color. The red, green, and blue cells develop selectivity to a view of, respectively, the bust, the head, and the face.
(3.3 MB MOV)

Video S2 Motorbike-Feature Learning The red cell becomes selective to the front part of a motorbike, while the green and blue cells both become selective to the wheels.
(6.8 MB MOV)

Video S3 Mixed Case The training set consisted of 200 face pictures, 200 motorbike pictures, and 200 background pictures. Notice that the red cell becomes selective to faces and the blue cell to heads, while the green cell illustrates how a given feature (round shape) can be shared by two categories.
(7.6 MB MOV)

## Abbreviations

IT: inferotemporal cortex
RBF: radial basis function
ROC: receiver operator characteristic
STDP: spike timing dependent plasticity
